# Lizards Cooperatively Tunnel to Construct a Long-Term Home for Family Members

**DOI:** 10.1371/journal.pone.0019041

**Published:** 2011-05-11

**Authors:** Steve McAlpin, Paul Duckett, Adam Stow

**Affiliations:** Department of Biological Sciences, Macquarie University, North Ryde, Australia; Arizona State University, United States of America

## Abstract

Constructing a home to protect offspring while they mature is common in many vertebrate groups, but has not previously been reported in lizards. Here we provide the first example of a lizard that constructs a long-term home for family members, and a rare case of lizards behaving cooperatively. The great desert skink, *Liopholis kintorei* from Central Australia, constructs an elaborate multi-tunnelled burrow that can be continuously occupied for up to 7 years. Multiple generations participate in construction and maintenance of burrows. Parental assignments based on DNA analysis show that immature individuals within the same burrow were mostly full siblings, even when several age cohorts were present. Parents were always captured at burrows containing their offspring, and females were only detected breeding with the same male both within- and across seasons. Consequently, the individual investments made to construct or maintain a burrow system benefit their own offspring, or siblings, over several breeding seasons.

## Introduction

Cooperative behaviour and social aggregations are relatively common in many animal groups, but rare in lizards, a large and otherwise behaviourally diverse group [1]. Mate fidelity is another trait that is uncommon in lizards [2]. Within social groups, cooperation is widely considered to be facilitated by genetic relatedness, and as such thought to have evolved in groups of related individuals [3]. In addition, breeding males are expected to invest more in their offspring as their certainty of paternity increases [4]. These predictions may explain the rarity of cooperative behaviours in lizards and their lack of investment in long-term home construction for groups of individuals.

Several lizard species belonging to the closely allied *Egernia* and *Liopholis* genera have kin-based sociality, a trait that is considered pleisomorphic to the group [5]. The only other lizard demonstrated to live in groups of related individuals is *Xantusia vigilis* [6]. Within the *Liopholis* and *Egernia* genera, species that form long-term groups tend to aggregate in pre-existing retreat sites, mostly rock crevices [2]. Although these species are characterised by groups that consist of close kin levels of polygamy vary both within and across species [2], [7]. One species, *Liopholis kintorei* constructs and maintains an interconnected network of tunnels within which it aggregates [2]. We have measured these spanning 13 meters across and with up to 20 entrances. Groups of individuals living within these consist of adults and immature lizards with overlapping generations. *Liopholis kintorei* is viviparous with 1–7 offspring produced annually [2]. The tunnels provide protection from predators and the extreme thermal environment in the region [2] and construction and regular maintenance must require a large investment of time and energy.

Here we evaluate the longevity of these burrow systems, whether multiple individuals construct and share tunnels, the relatedness among group members and their dispersal characteristics. Because kin-based sociality is an ancestral trait [5], cooperation among close kin would suggest that in this case inclusive fitness benefits have led to this behaviour. Furthermore, this would provide the first example of lizards constructing a long-term home for family members.

## Results

### Field observations

**Figure 1:**
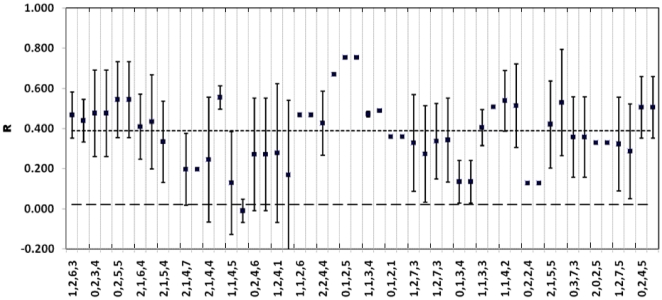
Relatedness composition within burrow systems. For each of 26 burrow systems two sets of relatedness data (R) are given, the average R±1 s.d between all lizards captured at that burrow and, in the next column to the right, R±1 s.d between the immature lizards. The average relatedness among all 120 genotyped individuals is shown by the lower horizontal line (R = 0.021), the upper horizontal line shows the average within-group relatedness (R = 0.371). Along the x-axis, for each group a series of numbers are given separated by commas. From bottom to top these are; the number of adults, age-cohorts of immature lizards, total group size of sampled individuals and the minimum number of years for which the burrow has been continuously occupied.

Adult and immature individuals use, share and maintain more than one tunnel in the system. This was directly observed and was evident from fresh tracks left by different sized individuals at tunnel openings. Tunnels are mostly excavated and maintained by adults and immature lizards contribute small ‘pop’ holes to the network. These are too narrow to be maintained by adult individuals. On average, only 6% of tunnels within a burrow system became disused each year. From the first record of the 26 burrow systems, the average (± s.d.) period of continuous occupancy in years is 4.04±1.43 (Fig. 1), each of these burrow systems had annual breeding success, so this time period represents 4 age cohorts of offspring. High philopatry of immature lizards to their natal burrow system was demonstrated by the genetic data.

### Parentage and relatedness analysis

**Figure 2:**
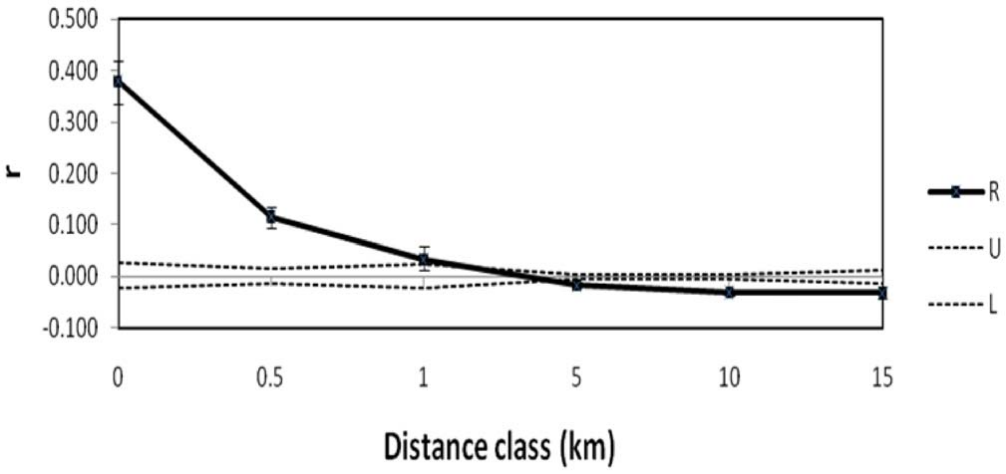
The distribution of genotypic similarity (r) with geographic distance (kilometres) for immature lizards. The solid line tracks relatedness, dashed lines represent the upper (U) and lower (L) 95% confidence interval around random expectations while bars around R show the 95% confidence interval around this estimate determined by bootstrapping. The distance class of 0 kilometres contains the r estimate among individuals sharing the same burrow system.

Polygynous males were detected with 40% of the male parents siring offspring to different females, and these were each located in different burrow clusters (separated by 18–179 meters). Juveniles from a single breeding season that were assigned the same mother show an absence of multiple paternity, and all females for which more than one age cohort of offspring were identified had bred with the same male across more than one breeding season. Only a single female was located in a different burrow system to one of its offspring (67 meters separate). As a consequence, groups of immature lizards only contained full siblings in 18 of 24 burrow systems where more than one immature lizard was sampled, and 12 of these contained siblings of more than one age cohort (Fig. 1). A low level of dispersing immature lizards is also demonstrated by the spatial distribution of relatedness. Levels of relatedness among immature lizards sharing the same tunnel system was high (mean relatedness ± s.d.; 0.446±0.123) and significantly greater than the relatedness among lizards located in different tunnel systems, even those located within 500 meters of each other (Fig. 2). Parents were always captured in a burrow containing their offspring and burrows in relatively close proximity (<500 meters) could contain immature lizards that shared the same father.

## Discussion

*Liopholis kintorei* cooperates to construct a burrow system that houses close kin (Fig. 1). Furthermore, the investment in time and energy that adult *L. kintorei* make towards creating this unique and elaborate tunnel system is realized by the high probability that it protects their offspring [4], [7], providing a unique example of parental care in lizards. For *L. kintorei* the relative importance of direct and indirect benefits of tunnel excavation and maintenance have not been estimated. Additionally, low levels of dispersal can increase competition between relatives [8]. Nonetheless, in *L. kintorei* there must be a net benefit to these behaviours and because they evolved in groups of close kin [5], this strongly suggests that inclusive fitness advantages played a role.

High natal philopatry of juvenile lizards has been associated with social aggregations of other members of the *Liopholis* and *Egernia* groups, and more recently, the unrelated lizard *Xantusia vigilis* [2], [6]. Indirect parental care in the form of reduced intraspecific aggression may offer a selective advantage to low juvenile dispersal [6], [9]. Our data on *L. kintorei* suggest that whatever the benefits of low juvenile dispersal are, they have driven the provisioning of an extensive burrow system in the absence of other retreat sites. Interestingly, two sympatric congeners *L. inornata* and *L. striata* appear to have lost their sociality and are largely solitary burrowers [2], [5].

For long-term social aggregations home sites need to be defendable and within foraging distance of a reliable food source [10]. The long-term stability of burrow systems constructed by *L. kintorei* implies that they may defend their home. The principle food source for *L. kintorei* is the termite *Drepanotermes perniger* [11] which has a naturally patchy distribution and burrow systems appear to be constructed in close proximity to the termite mounds. While the presence of a reliable food source in this arid environment seems necessary to support social aggregations of *L. kintorei*, it is unlikely to explain the evolution of these social groupings.

In mole rats the food-aridity hypothesis proposes that less rainfall is associated with increasing sociality because food resources become patchier [12]. Patchy food resources may select for individuals to aggregate close to food resources and cooperate because of the costs involved with dispersal and burrowing. However, the origin of the *Liopholis* lineage in mesic regions [13] and apparent loss of sociality for several species of *Liopholis* that occupy arid areas suggests that the food-aridity hypothesis may not explain social aggregations in *L. kintorei*. Furthermore, there are several *Egernia* species in arid regions that are primarily rock-dwelling (e.g. *E. stokesii*) and therefore have less choice when it comes to the location of their housing but still live in large kin-based social groups [2]. Nonetheless, the distribution and abundance of the primary food source for *L. kintorei* may influence aspects of their sociality. For example, it would be of interest to explore whether the proximity and longevity of termite colonies are associated with group sizes of *L. kintorei* and the length of time for which they occupy their tunnel systems.

The construction and maintenance of a long-term family home occurs in many other taxa, in vertebrates there are examples from most phyla, though it appears most prevalent in birds and mammals [4]. However, this form of parental care and cooperation to construct housing where ones offspring and siblings mature was, until now, unknown among lizards, a group containing at least 5000 species [14]. We have identified inclusive fitness benefits of this behaviour in *L. kintorei*, which, given the few examples of sociality in lizards, would also seem to explain its rarity.

## Materials and Methods

### Field collections

The study was conducted at Uluru – Kata Tjuta National Park, Northern Territory, Australia. From 1999 to 2009 monitoring was carried out once a year between September and April when lizards are most active. Monitoring consisted of searching for new burrow systems and inspecting all previously located burrow systems. For each burrow system the number of entrances and spatial organisation were recorded. Activity levels were noted by recording track activity and the presence of any fresh adult and immature lizard scats in their latrine area. Thirty hours of observations were carried out September to December 1996 within a raised hide located 8 meters from a burrow system containing an adult pair and 4 immature lizards. During this time burrowing activity, lizard locations and interactions were recorded. Trapping was undertaken during the summer activity periods at spatially discrete burrow systems distributed across 45 km^2^. Tissue biopsies were taken from the tail tips of 31 adult and 89 juveniles, with groups of individuals (mean group size ± SD; 4.19±1.67) sampled from 26 burrow systems. Sexing of adult individuals was carried out by visual appraisal. All methods involving *L. kintorei* were carried out in accordance with a protocol considered and approved by Parks Australia and the Macquarie University Ethics Committee under the Animal Research Authority 2008/025.

### Genotyping

Total DNA was extracted from 120 tissue samples using a salting-out protocol [15] and genotyped by amplifying seven microsatellite loci, ECU1, 2, 3 [16] and EST 1, 2, 9, 12 [17]. Numbers of alleles at these loci ranged from 8 to 24 and analysis of data from adults using the software GENEPOP 3.0.1 [18] showed that none of the loci significantly deviated from Hardy-Weinberg or linkage equilibrium. The combined non-exclusion probability for siblings was 0.0004, calculated using CERVUS 3.0.3 [19].

### Analysis of Relatedness

The maximum likelihood method of CERVUS 3.0.3 and COLONY 2.0.0.1 were used to assign parents to offspring [19]–[20]. All adult individuals (>165 mm SVL [2]) were included as candidate parents. Simulations for CERVUS were run with: 10000 cycles, 50% of candidate parents sampled, 100% of loci typed and a genotyping error rate of 1%. For our COLONY analysis we carried out a full-likelihood approach and allowed both males and females to be polygamous, and therefore the assignment of half siblings. We carried out a long-run with medium likelihood precision and no sib-ship prior. We used the same error rates as the analysis with CERVUS. Parental assignments were accepted if the candidate was not genetically incompatible at more than one locus and could be the parent with 80% or 95% confidence using CERVUS and that these assignments were compatible with those calculated using COLONY at p>0.8. COLONY identified groups of half and full siblings, even if one or both parents were not sampled. These sibling groups were accepted at p>0.8. In addition, the relatedness between individuals was estimated from allele frequency data obtained from all 120 samples. In order to calculate average levels of relatedness for individuals sampled within the same burrow complex we used a likelihood approach with KINGROUP 2.0 [21]. Dispersal patterns were inferred by examining the geographical structuring of relatedness using spatial autocorrelation analysis. Relatedness estimates calculated in GenAIEx 6.0 [22], were analysed at several distance classes. For each distance class, the significance of any deviation from zero was assessed by 999 permutations [22] and the 95% confidence intervals around relatedness were obtained via bootstrapping 999 times. Distance bins were chosen to estimate relatedness within a burrow system (distance = 0), among individuals sampled in different burrow systems that were within 0.500 kilometres of each other, and for individuals located between 0.500 and 0.999 kilometres and 1.000 meters to 14.000 kilometres of each other.
